# Individualised Estimation of Quality-adjusted Survival Benefit and Cost-effectiveness of Proton Beam Therapy in Intermediate-stage Hodgkin Lymphoma

**DOI:** 10.1016/j.clon.2023.01.007

**Published:** 2023-05

**Authors:** D.A. Jones, P. Candio, R. Shakir, J. Ramroth, J. Wolstenholme, A.M. Gray, D.J. Cutter, G. Ntentas

**Affiliations:** ∗ Nuffield Department of Population Health, University of Oxford, Richard Doll Building, Old Road Campus, Oxford, UK; † Oxford Cancer and Haematology Centre, Oxford University Hospitals NHS Foundation Trust, Oxford, UK; ‡ Department of Medical Physics, Guy's and St Thomas' NHS Foundation Trust, London, UK; § School of Biomedical Engineering and Imaging Sciences, King's College London, London, UK; ¶ Institute of Applied Health Research, University of Birmingham, Birmingham, UK; || Department of Economics and Management, University of Trento, Trento, Italy

**Keywords:** Cost-effectiveness, Hodgkin lymphoma, modelling, proton beam therapy, quality-adjusted life years

## Abstract

**Aims:**

Radiotherapy for Hodgkin lymphoma leads to the irradiation of organs at risk (OAR), which may confer excess risks of late effects. Comparative dosimetry studies show that proton beam therapy (PBT) may reduce OAR irradiation compared with photon radiotherapy, but PBT is more expensive and treatment capacity is limited. The purpose of this study is to inform the appropriateness of PBT for intermediate-stage Hodgkin lymphoma (ISHL).

**Materials and methods:**

A microsimulation model simulating the course of ISHL, background mortality and late effects was used to estimate comparative quality-adjusted life years (QALYs) lived and healthcare costs after consolidative pencil beam scanning PBT or volumetric modulated arc therapy (VMAT), both in deep-inspiration breath-hold. Outcomes were compared for 606 illustrative patients covering a spectrum of clinical presentations, varying by two age strata (20 and 40 years), both sexes, three smoking statuses (never, former and current) and 61 pairs of OAR radiation doses from a comparative planning study. Both undiscounted and discounted outcomes at 3.5% yearly discount were estimated. The maximum excess cost of PBT that might be considered cost-effective by the UK's National Institute for Health and Care Excellence was calculated.

**Results:**

OAR doses, smoking status and discount rate had large impacts on QALYs gained with PBT. Current smokers benefited the most, averaging 0.605 undiscounted QALYs (range –0.341 to 2.171) and 0.146 discounted QALYs (range –0.067 to 0.686), whereas never smokers benefited the least, averaging 0.074 undiscounted QALYs (range –0.196 to 0.491) and 0.017 discounted QALYs (range –0.030 to 0.086). For the gain in discounted QALYs to be considered cost-effective, PBT would have to cost at most £4812 more than VMAT for current smokers and £645 more for never smokers. This is below preliminary National Health Service cost estimates of PBT over photon radiotherapy.

**Conclusion:**

In a UK setting, PBT for ISHL may not be considered cost-effective. However, the degree of unquantifiable uncertainty is substantial.

## Highlights

• Proton beam therapy offers dosimetric advantage over photon radiotherapy.
• Lifetime modelling needed to estimate comparative benefits and cost-effectiveness.
• Baseline risks such as smoking increased quality adjusted survival benefit.
• Proton beam therapy unlikely to be cost-effective in UK under NICE threshold.
• There is a large amount of unquantifiable uncertainty when using modelling approach.

## Introduction

Radiotherapy is commonly used in the treatment of stage I and II Hodgkin lymphoma [1,2]. Long-term follow-up studies have, however, shown an associated increased risk of late effects, including second primary cancers and cardiovascular disease (CVD) [3,4]. These risks are stage- and patient-specific and depend on the distribution of disease, the patient age at treatment, sex, underlying health and the incidental radiation dose received by adjacent organs at risk (OAR) [[5], [6], [7], [8], [9], [10], [11]].

To minimise these risks, there is great interest in the use of proton beam therapy (PBT), which has been shown to reduce incidental irradiation compared with conventional photon radiotherapy in comparative planning studies [[12], [13], [14]]. However, these studies show large variation in dosimetry benefit across patients due to heterogeneity in anatomy and disease location [15]. In tandem with the high costs and capacity constraints of PBT, this illustrates the need to identify which Hodgkin lymphoma patients would benefit the most. It must also be determined whether the health-economic benefit from PBT is large enough to warrant the extra financial cost of PBT or, depending on the healthcare system, the use of limited PBT capacity for this indication at the cost of displacing the treatment of other cancer types. Converting dosimetric benefits into quality-adjusted life years (QALYs) provides an understandable and comparable measure by which the relative effectiveness and cost-effectiveness of PBT can be evaluated in clinical practice and for healthcare system planning.

In this study, through the use of an individual patient-level state-transition (microsimulation) model, we estimated the difference in expected QALYs and future healthcare costs between PBT and conventional photon radiotherapy for patients with chemo-responsive intermediate-stage Hodgkin lymphoma (ISHL) following positron emission tomography/computed tomography (PET/CT) response-adapted treatment. We applied our model to a clinically relevant population of illustrative patients, differing by age at treatment, sex, smoking status and radiotherapy modality. For the latter we used normal tissue radiation doses from a comparative planning study of 61 ISHL patients treated with pencil beam scanning PBT and re-planned with volumetric modulated arc therapy (VMAT), both in deep-inspiration breath-hold (DIBH) [16]. For each illustrative patient, we also calculated the maximum cost difference between PBT and VMAT necessary for PBT to be considered cost-effective in a UK setting, and compared this to currently available cost estimates.

## Materials and Methods

### Model Overview

A previously developed microsimulation model was used to estimate long-term outcomes in this study. Below we provide an overview of the model, with a fuller description given in a previous publication and in the supplementary material (Appendix 1) [11].

The model simulates the course of a patient's health over time, as represented by a set of health states. Time is discretised into yearly cycles, with the patient transitioning between health states at the end of each cycle, according to a set of time-specific transition probabilities. The simulated patient is assumed to be treated with an initial four cycles of ABVD chemotherapy followed by 30 Gy of radiotherapy, based on the standard therapy PET-negative arm of the H10U trial [17]. The model then explicitly simulates the recurrence of Hodgkin lymphoma, as well as excess second primary breast and lung cancer, coronary heart disease (CHD) and ischaemic stroke caused by irradiation of OAR. Each of these five diseases (i.e. Hodgkin lymphoma or one of the four late effects) forms an independent sub-module, allowing a patient to experience multiple diseases independently. Death could occur from one of the five diseases or from background all-cause mortality, with age- and sex-specific rates derived from Office for National Statistics mortality statistics for England and Wales [18].

Although we assume initial disease control and hence the risk of first relapse to be equivalent regardless of whether the patient was treated with conventional photon radiotherapy or PBT, the course of Hodgkin lymphoma is still explicitly modelled as it is a competing risk of death. The Hodgkin lymphoma sub-module is made up of five health states: remission, first relapse, second relapse, cured and dead from Hodgkin lymphoma. The annual probability of first relapse in the initial 5 years following the start of the model simulation were derived from the standard therapy (4 × ABVD chemotherapy plus 30 Gy radiotherapy) of the PET-negative arm of the H10U trial [17]. Simulated patients who did not suffer a first relapse within 5 years progressed to the cured state. After first relapse, patients were assumed to undergo salvage treatment with high-dose chemotherapy followed by autologous stem cell transplantation, with the probability of suffering a second relapse over the following 3 years derived from the Brentuximab Vendotin consolidation arm of AETHERA trail [19]. Finally, following a second relapse, the 10-year annual probability of death from Hodgkin lymphoma was derived from a single-centre study of outcomes after autologous stem cell transplantation relapse in the modern therapeutic era [20]. Patients transitioned to the cured state if they did not die during this 10-year at-risk period. Full details of the transition probabilities and the methods used in their derivation are provided in the supplementary material (Appendix 1, Appendix 5 Table 1).

**Table 1:** Average differences in organ at risk (OAR) mean doses between proton beam therapy and volumetric modulated arc therapy for all 61 comparatively planned intermediate-stage Hodgkin lymphoma patients and for indicative patient subgroups

OAR dose metric	All patients Average dose difference∗ (range) Gy	*P* Value	Average dose difference∗ by patient subgroup (Gy)											
			Longitudinal overlap CTV/heart			CTV inferior extension versus T7			CTV inferior extension versus LMSCA			Axillary involvement		
			<40% overlap (*n* = 42)	≥40% overlap (*n* = 19)	*P* Value	At or above T7 (*n* = 20)	Below T7 (*n* = 41)	*P* Value	Above (*n* = 13)	Below (n = 48)	*P* Value	Yes (*n* = 27)	No (*n* = 34)	*P* Value
MHD	–0.5 (–12.5 to 5.8)	0.64	0.7	–3.0	<0.01	1.0	–1.2	<0.01	1.2	–0.9	<0.001	–0.3	–0.6	0.66
MDCCA	1.5 (–1.6 to 7.9)	0.02	1.4	1.8	0.16	1.5	1.5	0.96	1.2	1.6	0.14	1.2	1.8	0.04
MLD	–2.2 (–6.7 to 0.4)	<0.001	–1.8	–3.0	<0.01	–2.0	–2.3	0.38	–1.8	–2.3	0.11	–2.6	–1.8	0.03
MBD	–1.0 (–6.0 to 1.1)	0.01	–0.7	–1.6	0.08	–0.8	–1.1	0.53	–0.6	–1.0	0.25	–1.5	–0.4	<.01

CTV, clinical target volume; LMSCA, left main stem coronary artery; MBD, mean breast dose to bilateral breast tissue; MDCCA, mean dose to the common carotid arteries; MHD, mean heart dose to the whole heart; MLD, mean lung dose to the whole lungs; T7, seventh thoracic level.

∗ Negative values indicate that proton beam therapy reduced the organ dose compared with photon radiotherapy.

Each late effect disease sub-module consists of three health states: no disease, disease, and dead from disease. Baseline yearly age- and sex-specific incidence and case-fatality rates, which determine the probability of transitioning from no disease to disease and disease to dead from disease, respectively, were abstracted from the PRIMEtime model, where they were derived from UK cohort studies and/or administrative healthcare datasets [[21], [22], [23]]. These rates were adjusted for smoking status, with the methodology outlined in the supplementary material (Appendix 1). In the model, patient-specific excess incidence rates for each of the diseases were calculated through modification of the smoking status-, age- and sex-specific incidence rates. This was carried out by calculating the patient's excess relative risks for the four late effects using their OAR dose metrics, namely mean dose to the breast tissue (MBD), lungs (MLD), heart (MHD) and common carotid arteries (MDCCA), linked to published estimates of the excess relative risk per Gy of radiation received by the respective OAR. The unadjusted rates were then subtracted to give the excess rate. The estimates of excess relative risk per Gy taken from previously published studies in Hodgkin lymphoma survivors are given in the supplementary material (Appendix 5 Table 2).

**Table 2:** Quality-adjusted life year (QALY) results by age, sex, smoking status and discount (3.5% or 0%) for all 61 comparatively planned intermediate-stage Hodgkin lymphoma patients

Discount	Sex	Age	QALYs PBT	QALYs VMAT	Difference
Undiscounted	Female	Never smokers, mean (range)			
		20	48.8 (48.4–49.0)	48.7 (48.2–49.1)	0.10 (–0.05–0.49)
		40	32.2 (32.1–32.4)	32.2 (31.9–32.4)	0.07 (–0.02–0.25)
		Former smoker, mean (range)			
		20	48.2 (47.6–48.7)	48.0 (47.1–48.6)	0.23 (–0.08–0.82)
		40	31.7 (31.2–32.1)	31.5 (30.7–32.0)	0.23 (–0.04–0.67)
		Current smoker, mean (range)			
		20	46.9 (45.6–47.9)	46.4 (44.5–47.7)	0.54 (–0.19–1.54)
		40	30.5 (29.3–31.3)	29.9 (28.1–31.0)	0.58 (–0.15–1.50)
	Male	Never smokers, mean (range)			
		20	46.2 (45.9–46.6)	46.2 (45.7–46.5)	0.06 (–0.20–0.43)
		40	29.9 (29.7–30.0)	29.8 (29.5–30.0)	0.07 (–0.05–0.27)
		Former smoker, mean (range)			
		20	45.6 (44.9–46.2)	45.3 (44.3–46.1)	0.24 (–0.23–1.03)
		40	29.2 (28.7–29.7)	29.0 (28.1–29.5)	0.27 (–0.08–0.89)
		Current smoker, mean (range)			
		20	44.0 (42.6–45.3)	43.4 (41.5–45.0)	0.60 (–0.34–2.17)
		40	27.8 (26.5–28.8)	27.1 (25.3–28.5)	0.67 (–0.21–2.15)
3.5% discount	Female	Never smokers, mean (range)			
		20	21.3 (21.3–21.4)	21.3 (21.2–21.4)	0.02 (–0.01–0.09)
		40	17.2 (17.2–17.3)	17.2 (17.1–17.3)	0.02 (–0.00–0.07)
		Former smoker, mean (range)			
		20	21.3 (21.2–21.3)	21.2 (21.1–21.3)	0.04 (–0.01–0.14)
		40	17.1 (17.0–17.2)	17.0 (16.8–17.2)	0.07 (–0.01–0.20)
		Current smoker, mean (range)			
		20	21.1 (20.9–21.2)	21.0 (20.7–21.2)	0.09 (–0.03–0.25)
		40	16.7 (16.4–17.0)	16.5 (16.0–16.9)	0.18 (–0.05–0.47)
	Male	Never smokers, mean (range)			
		20	20.9 (20.9–21.0)	20.9 (20.8–21.0)	0.01 (–0.03–0.06)
		40	16.6 (16.5–16.6)	16.55 (16.5–16.6)	0.02 (–0.02–0.08)
		Former smoker, mean (range)			
		20	20.8 (20.7–20.9)	20.8 (20.6–20.9)	0.04 (–0.04–0.16)
		40	16.4 (16.2–16.5)	16.3 (16.0–16.5)	0.08 (–0.02–0.26)
		Current smoker, mean (range)			
		20	20.6 (20.3–20.8)	20.5 (20.2–20.7)	0.10 (–0.06–0.35)
		40	15.9 (15.5–16.3)	15.7 (15.1–16.2)	0.21 (–0.07–0.69)

PBT, proton beam therapy; VMAT, volumetric modulated arc therapy.

Life years are accrued for every cycle in which the patient is alive. QALYs are calculated by adjusting life years by age- and disease-specific EQ-5D utility values. Age-specific EQ-5D utilities and decrements associated with each disease were taken from the Catalogue of EQ-5D Scores for the United Kingdom [24]. These can be found in the supplementary material (Appendix 5 ).

Although our model may be run probabilistically to produce a 95% confidence interval corresponding to known parameter uncertainty, due to the small intervals observed in our previous study, the model was run deterministically, using mean values for the parameters [11]. With this set of parameters, the life course of a patient is simulated 10 000 times and averaged to limit Monte Carlo error.

### Cost of Late Effects

In order to evaluate the possible cost-effectiveness of PBT, the previously developed model was augmented to also calculate UK National Health Service costs attributable to late effects. A systematic literature search was carried out to identify appropriate studies on which to base these costs. Details of the search and the selection of costs included in the model, as well as the costs themselves, are provided in the supplementary material (Appendix 2). Costs are reported for the year 2014/15.

### Comparative Dosimetry and Application of the Model

Lifetime QALYs and healthcare costs generated by the model are a function of the simulated patient's age at treatment, sex, smoking status and three or four OAR dose metrics, namely MLD, MHD, MDCCA and, for females, MBD. In this analysis, we used the mean organ doses of 61 ISHL patients from a planning study that compared radiation doses from patients treated with pencil beam scanning PBT in DIBH and re-planned with VMAT in DIBH [16]. The planning method used for most patients was butterfly VMAT (BVMAT), but for more complex volumes additional plans were produced using BVMAT with additional partial or full arcs and a ‘best plan’ chosen based on planning target volume coverage and OAR doses. Full details of patient contouring, planning and dosimetry analysis can be found in the study publications [12,16]. In the Results section, we provide tabulations of patient dosimetry for these 61 patients.

To provide a comprehensive range of clinically relevant patient presentations and a wide range of dosimetric differences, we used the 61 mean organ dose pairs in combination with two ages at treatment (20 and 40 years), each sex and three smoking statuses (never, former and current). For the female combinations, only OAR dose metrics for the 40 patients who were female, and therefore had MBDs, were used. Therefore, in total, 606 (2 × 3 × 40 + 2 × 3 × 61) distinct patient presentations resulting from the age, sex, smoking status and OAR radiation doses were run through the model, generating for each their expected difference in life years, QALYs and lifetime healthcare costs attributable to late effects between treatment with PBT and VMAT. The generation of the 606 illustrative patients is illustrated in the supplementary material (Appendix 3). The simulations were run both undiscounted and with a 3.5% annual discount rate applied to future health outcomes and costs, as recommended by the UK's National Institute for Health and Care Excellence (NICE) for cost-effectiveness analysis.

PBT is not currently commissioned for the treatment of lymphoma in adults in the UK, due to a lack of evidence regarding clinical and cost-effectiveness [25]. In England, NICE makes treatment reimbursement recommendations based on consideration of cost-effectiveness judged according to the incremental cost-effectiveness ratio (ICER). This is the ratio of the difference in costs and QALYs between the treatment and its next best comparator. The ICER is then compared to NICE's willingness-to-pay threshold, which is stated to be in the range of £20 000–30 000 per QALY. Due to the nascency of PBT in the UK, published peer-reviewed estimates of treatment cost do not currently exist, which precludes direct estimation of the ICER of PBT over VMAT. However, for a given willingness-to-pay threshold, it is possible to calculate the maximum cost of PBT over VMAT for PBT to be considered cost-effective. The rearrangement of the ICER equation underlying this estimation is given in the supplementary material (Appendix 4). Using the QALY and healthcare cost outcomes generated by our model, we calculated the maximum excess cost at which PBT might be considered cost-effective for each of the patient presentations, under the £30 000 per QALY willingness-to-pay threshold.

Finally, we investigated the results according to four pre-treatment indicators of difference in comparative dosimetry [16]. Three relate to improved cardiac sparing, of which two were explored in a prior comparative study: (i) the longitudinal overlap of the clinical target volume (CTV) and heart (dichotomised into above or below 40%); and (ii) CTV extension above or below the seventh thoracic level (T7). Additionally, the International Lymphoma Radiation Oncology Group guidelines identified (iii) CTV extension above or below the origin of the left main stem coronary artery (LMSCA) as another indicative factor [26]. And fourthly, relating to improved lung and breast sparing, irradiation or not of the axilla. We assessed whether these indicators translated into larger differences in QALYs and greater cost-effectiveness of PBT.

## Results

### Comparative Dosimetry

Across all 61 comparatively planned ISHL patients, PBT modestly reduced the MHD, MLD and MBD by, on average, –0.47, –2.18 and –0.97 Gy, respectively, as shown in Table 1. Conversely, PBT on average increased MDCCA by 1.51 Gy. Table 1 also gives the average differences in mean doses for the dichotomies of the four indicators outlined above. For the three indicators relating to improved cardiac sparing, the longitudinal overlap of the CTV and heart produced the largest difference. Those with an overlap of <40% had on average a 0.66 Gy higher MHD with PBT, whereas for those with an overlap of ≥40%, PBT reduced MHD by 2.96 Gy. There was also a greater reduction in lung and breast doses with PBT for patients receiving radiation to the axilla.

### Quality-adjusted Life Years

**Fig 1:**
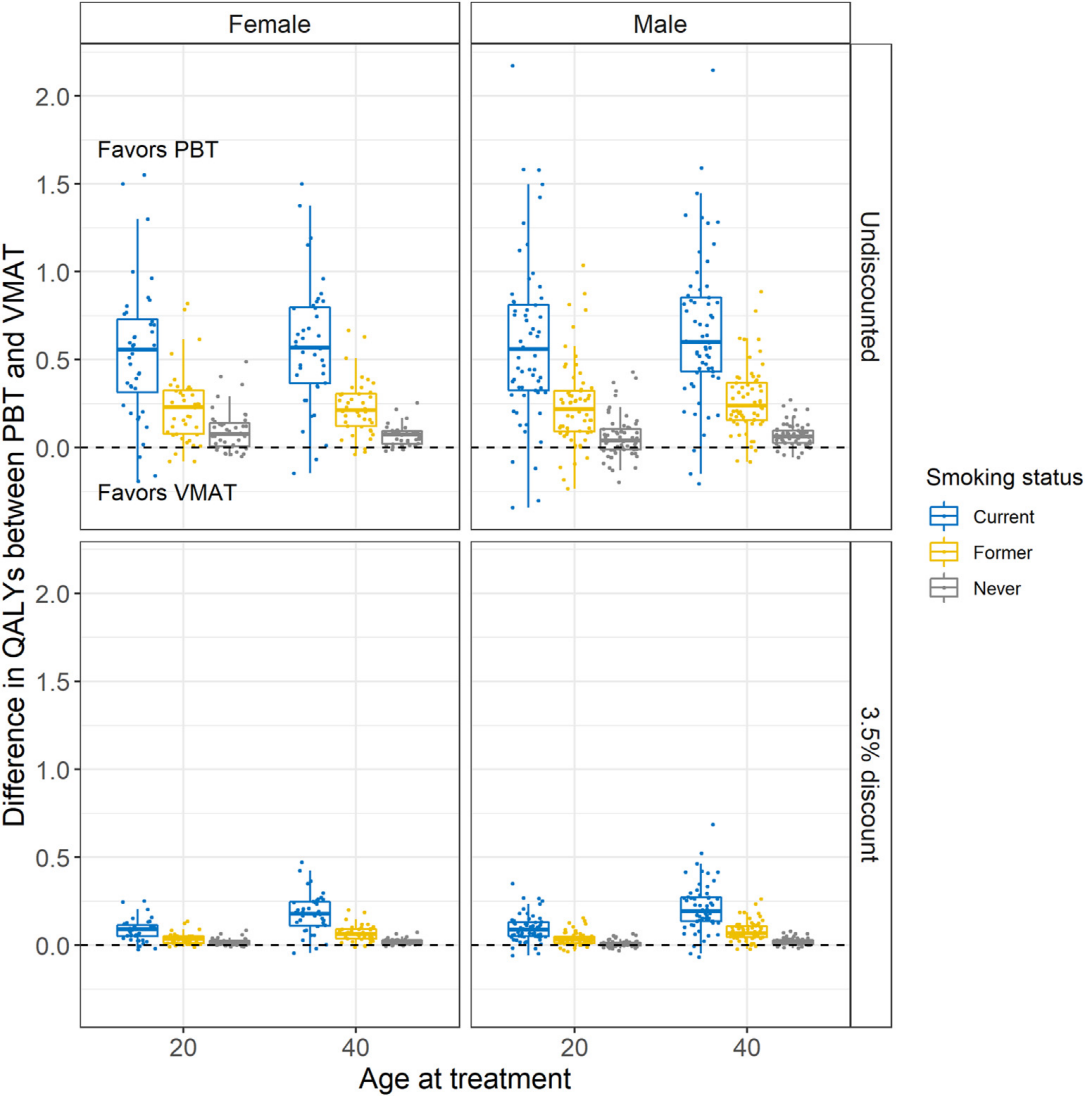
Box plots of the estimated difference in quality-adjusted life years (QALYs) between proton beam therapy (PBT) and volumetric modulated arc therapy (VMAT) by age, sex, smoking status and discount rate.

Nearly all of the 606 illustrative patients were estimated to gain an increase in QALYs with the use of PBT over VMAT. For only a small minority of the illustrative patients (63 of 606), whose mean radiation doses from VMAT were smaller for one or more organ, VMAT provided greater QALYs. Across all simulated patients, PBT provided a mean gain of 0.307 undiscounted (0.073 discounted) QALYs, ranging from –0.342 (–0.067) to 2.171 (0.686). Patient characteristics influenced the number of QALYs gained or lost with PBT, with smoking status, OAR doses and health discount rate having the largest impact. Table 2 provides the mean number of QALYs gained after treatment for PBT and VMAT by age at treatment, sex, smoking status and discount (3.5% or 0%) for the 61 OAR doses. Figure 1 illustrates the difference in QALYs for these same groupings.

Current smokers gained the most QALYs, averaging 0.605 undiscounted QALYs, ranging from –0.342 to 2.171 across the age, sex and OAR dose combinations. This average fell to 0.146 QALYs (range –0.067 to 0.686) when future health was discounted at a rate of 3.5%. In contrast, the benefit to the never-smoker patients was far lower. Undiscounted, the benefit averaged 0.074 QALYs (range –0.196 to 0.491), whereas with discounting the benefit averaged 0.017 (range –0.030 to 0.086). As shown in Figure 1, patient sex had minimal impact on the average QALYs gained with PBT. Similarly, age at treatment had little effect in the undiscounted analysis. However, with the application of the 3.5% to future health and cost outcomes, QALYs gained increased with older age at treatment, especially for current smokers. For instance, a 40-year-old male current smoker on average gained 0.213 QALYs compared with 0.096 QALYs for a 20-year-old male current smoker.

**Fig 2:**
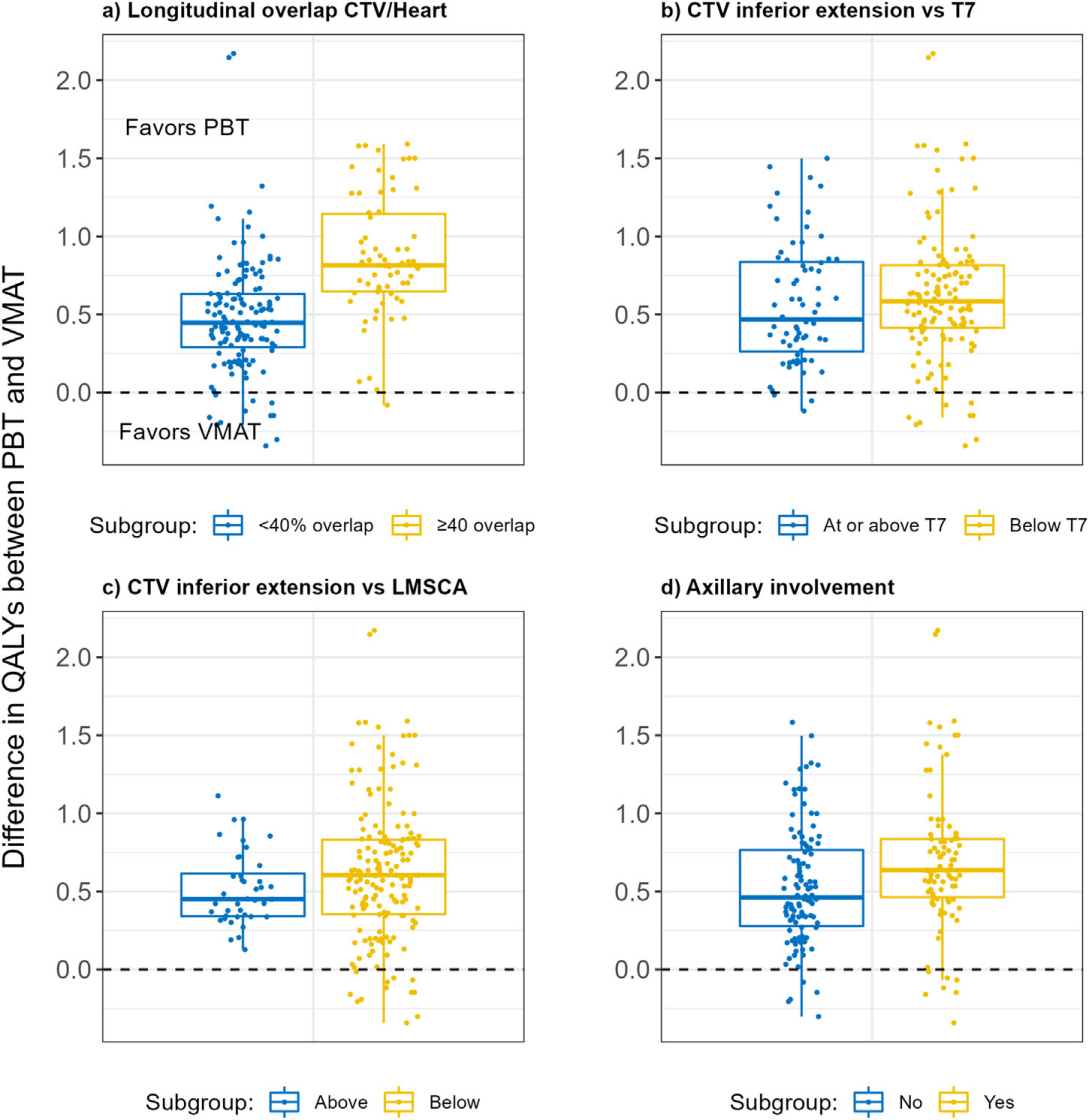
Box plots of the estimated difference in undiscounted quality-adjusted life years (QALYs) between proton beam therapy (PBT) and volumetric modulated arc therapy (VMAT) according to pre-treatment planning characteristics for all current-smoker illustrative patients. CTV, clinical target volume.

Visual exploration (Figure 2) of differences according to pre-treatment planning characteristics suggests the longitudinal overlap of the CTV to the heart, with a threshold of 40%, may be indicative of a higher QALY benefit. The mean undiscounted benefit in all current smokers with an overlap ≥40% was 0.866 QALYs compared with 0.478 QALYs in those with smaller or no overlap. In never smokers, the benefit was 0.141 QALYs and 0.041 QALYs, respectively. CTV below T7, CTV in relation to the origin of the LMSCA and axilla involvement appeared less predictive. The same pattern of results occurred when using the former-smoker illustrative patients.

### Maximum Additional Cost of Proton Beam Therapy to be Considered Cost-effective

**Fig 3:**
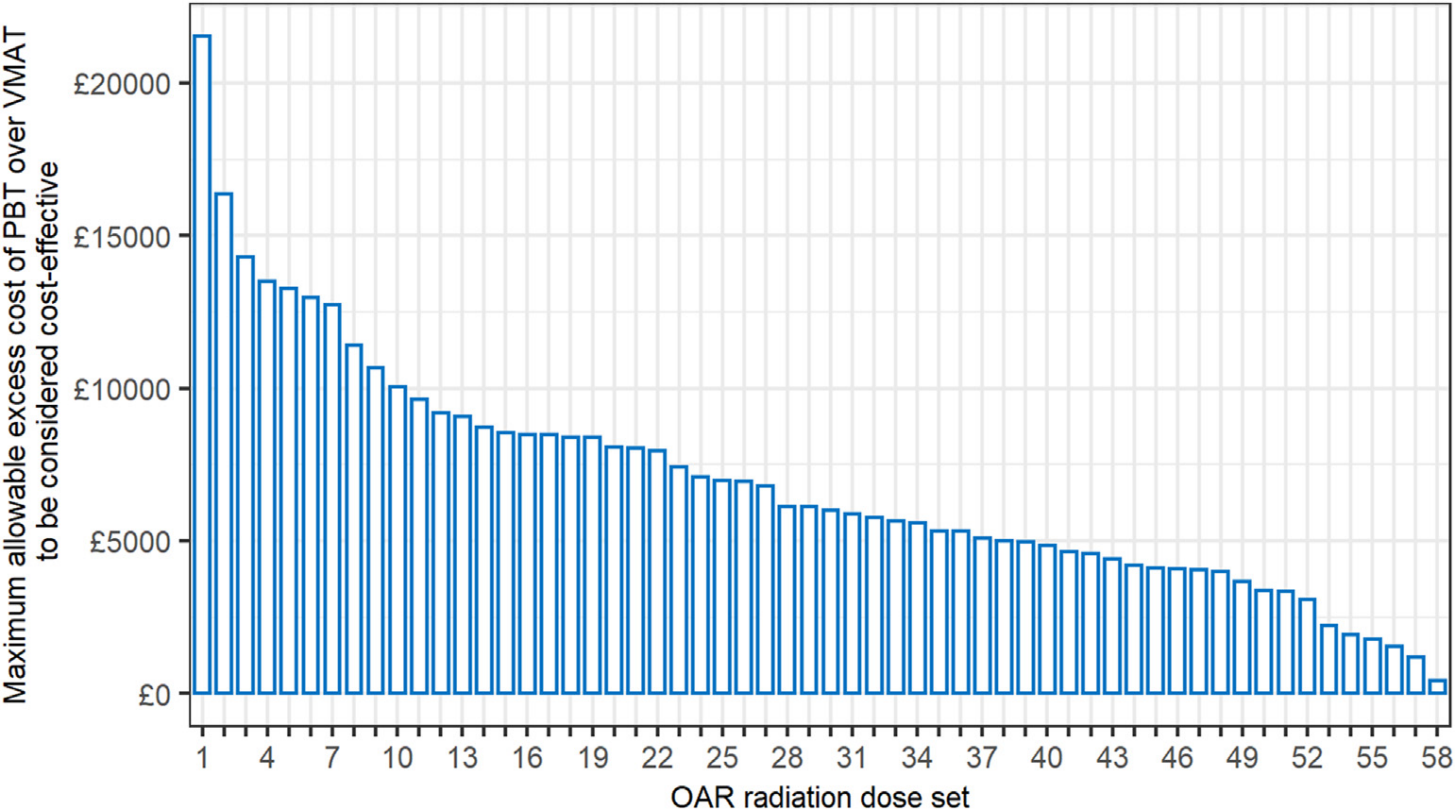
Distribution of the maximum allowable excess cost of proton beam therapy (PBT) over volumetric modulated arc therapy (VMAT) to be considered cost-effective for male current-smoker illustrative patients. Each column of the *x* axis, ordered from highest additional cost to lowest, constitutes one of the 58 (of the 61) organ at risk (OAR) radiation sets from the comparative planning study.

In line with the results for QALYs, smoking status had a significant effect on the maximum additional cost of PBT over VMAT to be considered cost-effective. Using the £30 000 cost-effectiveness threshold, the maximum allowable excess cost of PBT (compared with photon radiotherapy) averaged £4812 for the current-smoker illustrative patients compared with £645 for never smokers, but varied considerably, with a maximum of £21 538 and £3127, respectively. Figure 3 highlights this variation using the 40-year-old male current smoker as an example, with each column being one of the 58 different OAR dose sets from the planning study, excluding three (of the 61) OAR dose sets, which led to a reduction in QALYs with the use of PBT. Grouping by CTV and heart overlap, the maximum allowable excess cost of PBT under a £30 000 threshold for current smokers averaged £6593 for patients with an overlap ≥40% and £3886 for those with an overlap <40%. The equivalent accepted costs for never smokers were £993 and £426.

Full tabular results of the estimated difference in QALYs, difference in late effects-related healthcare costs and the maximum excess upfront cost of PBT to be considered cost-effective are provided in the supplementary material (Appendix 5 Table 8).

## Discussion

In terms of QALYs, our results show variation in the benefit estimated from using PBT. The benefit varied greatly according to OAR doses, which are a consequence of different disease distributions and patient anatomy. Smoking status also had a significant impact on the magnitude of the predicted benefit of PBT, through its influence on incidence rates of lung cancer, CHD and stroke, and current smokers received the greatest gains in QALYs. Age at treatment had an impact on QALYs gained when future health and costs were discounted, suggesting a greater absolute benefit of PBT at older ages at treatment. This is due to the interaction between the monotonically increasing incidence rates of cancers and CVD with age, and the increasing impact of discounting the further away from year of treatment. Therefore, although younger patients have a longer time at risk, late effects in the model are far more likely to occur in older age, by which time their net present impact has been substantially reduced by the discount factor. Sex had minimal impact on results, despite the added risk of radiation-induced breast cancer in females. This is due to the distribution of the age-specific annual rates for the three other late effects, with baseline incidence and case-fatality rates for lung cancer, CHD and ischaemic stroke being higher at younger ages for males than for females of the same age.

Few studies have looked at the cost and cost-effectiveness of PBT, and no peer-reviewed studies have been carried out in a UK setting for any cancer site [27]. Estimating the cost of PBT is a non-trivial exercise [28] and as yet there remains no National Health Service reference cost for the preparation and delivery of a fraction of PBT, nor any other published costing study. As such, we primarily present estimates of the maximum additional upfront cost difference at which PBT might be considered cost-effective under a £30 000 willingness-to-pay threshold. The UK's Department of Health does present a crude per patient incremental cost estimate compared with conventional photon radiotherapy in its strategic outline case for the development of a national PBT service in England [29]. At £34 359, this is far above the average of our individual patient maximum cost estimates. This suggests that PBT is currently unlikely to be considered cost-effective for the average ISHL patient in the UK setting. To our knowledge, only one other cost-effectiveness study of PBT in Hodgkin lymphoma, in a US setting, has been carried out. This study focused on the risk of CHD only. The results of the study, which also used comparative dosimetry, suggested that PBT is also unlikely to be cost-effective in a US setting [30].

Model-based selection of patients to novel radiotherapy technologies based on estimated comparative outcomes necessitates the generation of OAR dose estimates from dual treatment plans. However, generating dual treatment plans for all patients is time consuming and costly. To partially address that, pre-treatment predictive factors have been proposed for initial planning selection as a more pragmatic approach and, to this end, we assessed four predictive indicators previously identified [16,26]. The longitudinal overlap of the CTV in relation to the heart with a threshold of 40% seems to be predictive of benefit, aligning with findings of improved 30-year absolute mortality risk from CVD in the comparative planning study [16]. However, our sample of comparatively planned patients is small from a statistical perspective, and our synthetic generation of illustrative patients precluded the use of formal statistical testing of difference.

The range of doses from the comparative planning study, as shown in Table 1, encompass cases presented explicitly in the International Lymphoma Radiation Oncology Group and Particle Therapy Cooperative Group studies, as well as summary doses from other comparative planning studies [13,14,26,31], suggesting that this sample of patients is representative of the majority of possible disease presentations. Nonetheless, due to the rapid advancement of PBT techniques and natural lag in the publication of comparative planning studies, more advanced PBT techniques may now, and in the future, generate larger dosimetric differences between PBT and VMAT.

Our model accounts for the increased risk of the most clinically important late effects [3,4,32]. However, radiotherapy has been shown to increase the risk of a number of other rarer second malignancies, such as oesophageal and thyroid cancer, as well as other cardiovascular events, such as valvular heart disease and heart failure [[33], [34], [35], [36]]. Inclusion of all possible late effects is challenged by the availability of incidence and case-fatality data, but it should be noted that their omission may underestimate the total comparative benefit of PBT. This underestimation is likely to be small however, given our inclusion of the most important late effects in terms of mortality [37].

In model-based cost-effectiveness studies, joint parameter uncertainty is often propagated through probabilistic sensitivity analysis to give confidence intervals for the outcomes. This is an estimate of the joint known uncertainty in the input parameters. Although our model can facilitate this, the vast majority of our input parameters, taken from various studies, did not have published uncertainty measures, such as confidence intervals or standard deviations. Therefore, confidence intervals of parameter uncertainty for our health and cost outcomes would be artificially narrow, which could wrongly be interpreted as a large degree of certainty in our results. Furthermore, the complexity of modelling many diseases far into the future leads to a high degree of structural and methodological uncertainty, which is difficult to quantify [38]. Unquantifiable uncertainty is inherent in all research, and not limited to cost-effectiveness analysis. Nonetheless, appropriate caution in the results should be taken in light of these uncertainties.

To conclude, despite the limitations inherent in the use of models to predict future outcomes, it is only through studies such as ours that the predicted impact of novel radiotherapy technologies aimed at reducing the burden of late effects can be explored. The results from our study suggest that, taken as a whole, ISHL patients may only gain modestly from PBT in terms of QALYs. However, certain patients who are predisposed to poorer outcomes, such as smokers, or those with large difference in radiation dose to OARs, are predicted a larger gain in quality-adjusted life expectancy. Due to the additional cost of PBT and the inherent devaluation of future events resulting from the use of discounting in economic evaluations, our results suggest that PBT for ISHL is unlikely to be cost-effective for most ISHL patients in the UK.

## Funding

This research was supported by a 10.13039/501100000289Cancer Research UK Centres Network Accelerator Award Grant to the ART-NET Consortium (A21993) and a 10.13039/501100000289Cancer Research UK programme grant (C8225/A21133). RS is supported by a National Institute 10.13039/100005622for Health and Care Research Doctoral Studentship (NIHR300740). 10.13039/100015876GN was supported by an Early Career Postdoctoral Research Fellowship from the Nuffield Department of Population Health, 10.13039/501100000769University of Oxford and a National Institute 10.13039/100005622for Health and Care Research Clinical Lectureship (NIHR301261) funded by Health Education England (HEE). These funding bodies had no role in the collection, analysis or interpretation of the data, or in the writing of the report.

## Author Contributions

DJ, DC and GN are the guarantors of integrity of the entire study. DJ, PC, RS, JR, JW, AG, DC and GN were responsible for study concepts and design. DJ and GN were responsible for experimental studies/data analysis, statistical analysis and manuscript preparation. DJ, PC, RS, JR, JW, AG, DC and GN edited the manuscript.

## Conflicts of Interest

The authors declare no conflicts of interest.
