# Supplementary material for: Individualised Estimation of Quality-adjusted Survival Benefit and Cost-effectiveness of Proton Beam Therapy in Intermediate-stage Hodgkin Lymphoma
Source: Clin Oncol (R Coll Radiol). 2023 May;35(5):301–10. doi: 10.1016/j.clon.2023.01.007 (PMC11913763; doi:10.1016/j.clon.2023.01.007)
Supplement: Multimedia component 1 [file mmc1.docx]

Supplementary Material

Contents

[Appendix 1 - Model summary 2](#_Toc115861672)

[Intermediate-stage Hodgkin lymphoma sub-model 2](#_Toc115861673)

[Excess breast and lung cancer, coronary heart disease and ischaemic stroke sub-models 2](#_Toc115861674)

[Smoking status adjustment 3](#_Toc115861675)

[Dose-response relationships to predict late effects per Gray of radiation 4](#_Toc115861676)

[Appendix 2 – Cost of late effects 6](#_Toc115861677)

[Systematic search of costing studies for breast and lung cancer, CHD, and stroke 6](#_Toc115861678)

[Incorporation into model 10](#_Toc115861679)

[Appendix 3 – Deriving the maximum additional cost of PBT over photon-based radiotherapy for PBT to be considered cost-effective 12](#_Toc115861680)

[Appendix 3 – Generation of illustrative patients 13](#_Toc115861681)

[Appendix 4 – Additional tables 15](#_Toc115861682)

[Table 1: Health state transitions in the ISHL sub-model 15](#_Toc115861683)

[Table 2: Summary of the published dose-response relationships used to estimate risks of radiation-related second cancers, CHD and stroke 15](#_Toc115861684)

[Table 3: EQ-5D values from Sullivan et al (2011)(41) 16](#_Toc115861685)

[Table 4: Proportions of people who have never smoked, ex-smokers and smokers from the 2012 General Lifestyle Survey 16](#_Toc115861686)

[Table 5: Relative risk and 95% confidence interval for lung cancer, CHD, and stroke used to adjust the population incidence rates by smoking status 17](#_Toc115861687)

[Table 6: The costs of breast and lung cancer 17](#_Toc115861688)

[Table 7: The costs of CHD and stroke 18](#_Toc115861689)

[Table 8: Difference in QALYs, health care costs and maximum additional upfront cost of PBT to be considered cost-effective at £30,000 threshold 18](#_Toc115861690)

[Appendix 5 – Additional Figure 20](#_Toc115861691)

[Figure 1: Box plots of the estimated difference in undiscounted quality-adjusted life years between PBT and VMAT for former smokers across the four prognostic markers 20](#_Toc115861692)

[References 21](#_Toc115861693)

# Appendix 1 - Model summary

## Intermediate-stage Hodgkin lymphoma sub-model

The standard of care (four cycles of ABVD chemotherapy followed by 30Gy consolidative radiotherapy) arm of the H10U trial was used as the basis of risk of first relapse in this analysis.(1) A simulated patient is assumed to start in remission and have negative findings on PET after four cycles of ABVD chemotherapy (chemo-responsive). From this state patients are at risk of first relapse, with the yearly probability of relapse derived through reconstruction of individual patient time-to-event data using the Kaplan–Meier (KM) plot (Figure 4b) of progression-free survival (PFS) from the H10 trial publication.(2) Parametric survival models (Exponential, Weibull, Gamma, log-Normal, log-Logistic, Generalised gamma, Generalised F and Gompertz distributions) were fitted to the reconstructed data, with yearly transition probabilities derived from the best fitting parametric model, using Akaike and Bayesian information criteria.(3) This was the Generalized F distribution. Patients in the remission state were considered cured after 5 years due to the rarity of relapses past this point.(4)

It was assumed that all relapsed patients would receive salvage treatment with high‐dose chemotherapy followed by autologous stem cell transplantation (ASCT). Yearly probabilities of second relapse were estimated from the brentuximab vendotin consolidation arm of the AETHERA trial, using the same procedure as above.(5) After a second relapse, the yearly probability of death from HL was derived from the overall survival KM plot from a single centre study of outcomes after ASCT relapse in the era of brentuximab vedotin, and immune check point inhibitors.(6) This used the same methodology as previously described. After 10 years, if a patient has not died, they transition to the cured state, in which they are assumed to have the same background mortality rate as the general population, in addition to any increased risk from late-effects from radiotherapy.

## Excess breast and lung cancer, coronary heart disease and ischaemic stroke sub-models

Simple and generalisable disease sub-models were used to simulate the excess incidence and mortality of new primary breast and lung cancer, CHD and ischemic stroke over the patient's life course after undergoing consolidative radiotherapy.(7–10) These consisted of three health states; no disease, diseased, and dead from disease. It was assumed that the background incidence and progression of each late effect was similar to that of the general population, with incidence modified by the ERR/Gy from irradiation of the specific OAR.

Baseline rates (without irradiation) used for the probabilities of transitioning from no disease to diseased (incidence) and from diseased to dead from disease (case-fatality) were abstracted from the PRIMEtime model.(11) Full description and presentation of the data and methods have been published extensively.(12,13) Briefly, age- and sex-specific disease incidence rates were derived from UK cohort studies and/or administrative healthcare datasets and disease-specific mortality rates were taken from ONS cause-specific death registrations. Internally consistent age- and sex-specific case-fatality rates were then derived using the World Health Organisation’s DISMOD II program.

## Smoking status adjustment

Smoking is associated with an increased risk of lung cancer, coronary heart disease and stroke. This increase is especially substantial in lung cancer, with a large majority of cases being smoking-related. We, therefore, adjusted the population-based incidence rates, which are an aggregation of all smokers and non-smokers, into incidence for never, former and current smokers as follows:

1. Estimates of the prevalence of never, former, and current smokers in the UK population by age and sex were obtained from the 2012 General Lifestyle Survey.(14) These can be found in table S4.
2. Estimates of the relative risk of disease for lung cancer CHD and Stroke were identified from the Royal College of Physician’s “Hiding in plain sight: Treating tobacco dependency in the NHS” report, which is used by Public Health England.(15) Table S5 provides the estimates.
3. The yearly age- and sex-specific population-based incidence rates, relative risks, and prevalence data were used to calculate the incidence rate in non-smokers using the equation:

$${IRnonsmokers}_{dsi}= \frac{{IRpop}_{dsi}}{(1+ \sum_{k} {(RR}_{dsk}-1)*{Prev}_{ski})}$$

where,

${IRnonsmokers}_{dsi}$ = incidence rate for non-smokers in disease d, sex s, and age category i

${IRpop}_{dsi}=$Incidence rate for the population (all risk groups combined) in disease d, sex s, and age category i

$${RR}_{dsk}= relative risk for incidence in disease d, sex s, and smoking group k (k= 1: former smokers, 2: current smokers)$$

$${Prev}_{ski}= prevalence estimate in sex s,smoking group k, and age category i$$

1. Finally, the incidence rate for former and current smokers were calculated using the following formula:

$${IR}_{dski}={IRnonsmokers}_{dsi}* {RR}_{dsk}$$

where,

${IR}_{dski}$ = incidence rate in disease d, sex s, smoking group k, and age category i

Individualised excess incidence rates were calculated by first modifying the smoking staus-, age- and sex-specific incidence rates by the excess relative risk of disease, derived by inputting the specified MBD, MLD, MHD and MDCCA to the dose-response equations. The unadjusted rates were then subtracted. Additionally, the incidence and case-fatality rates were adjusted for their predicted 20-year trend.(12)

## Dose-response relationships to predict late effects per Gray of radiation

*Ischemic heart disease*

The dose response relationship for the incidence of CHD was taken from a case-control study of HL survivors as detailed in appendix Table 2.(10) This study gives an excess relative risk (ERR) per Gray (Gy) MHD by age at treatment in 3 groups (<28 years, 28 to 36 years and >36 years). Chemotherapy exposure was not taken into account.

*Stroke*

Standardised incidence ratios (SIRs) for the risk of stroke in a cohort of HL survivors treated with mantle field radiotherapy, were published by De Bruin et al.(16) A radiation dose-response relationship was not provided by the authors and, since the MDCCA for each patient in this study were unknown, a dose-response model could not be derived directly. However, Maraldo et al provided a retrospective dose reconstruction method for estimating MDCCA as a percentage of the prescribed dose for HL patients treated with mantle and involved field radiotherapy.(17) Since the cohort in the De Bruin et al study received predominantly mantle field radiotherapy, this method was used to estimate the average MDCCA for this cohort to derive an ERR per Gy of MDCCA.(16) The SIRs reported in the De Bruin et al study were divided by the estimated MDCCA in order to derive ERR per Gy of MDCCA for the risk of stroke.

Not all cases of stroke reported in the De Bruin et al study were first presentations of cardiovascular disease. Of the 65 stroke cases, 17 had developed prior heart disease. Prior heart disease was reported to increase the risk of stroke by a factor of 2.1 compared to having no prior heart disease. As the focus of our study was the first cardiovascular event, the ERR per Gy for stroke was thus scaled to account for a reduced risk of stroke as a first cardiovascular event. To derive the appropriate scale factor, it was assumed that 0.74X + 0.26 x 2.1X = Y, where X is the ERR per Gy for stroke without prior heart disease applicable to the 74% of patients who did not have prior heart disease, 2.1X is the ERR per Gy applicable to the 26% of patients who did have prior heart disease, and Y is the ERR per Gy for all patients in the study. It follows that X= Y/1.286, so the observed ERRs per Gy were divided by 1.286.

*Second breast and lung cancers*

Dose-response relationships for excess risks of second cancers due to radiotherapy were obtained from case-control studies in HL survivors and are shown in appendix Table 1.(7–9) Possible excess cancer risks due to chemotherapy were not taken into account, as data detailing the dose-response relationship is lacking. For breast cancer the ERR per Gy MBD was adjusted for the relative risk varying with age of exposure using data from two large cohort studies.(18,19) The case-control studies that produced the dose-response relationships utilised in the predictions presented these relationships in terms of estimated point dose at the site of the second cancer as the dose metric for exposure. However, point dose is not the most appropriate measure for predicting future risk as it is not known exactly where a future cancer may develop. We therefore used published dosimetry data to define the ratio between the mean dose at the point the cancer developed (as a percentage of prescribed dose)(7–9) to the mean organ dose (as a percentage of prescribed dose)(17) to derive an adjustment factor for the ERR per Gy used for the predictions (see appendix Table 2).

# Appendix 2 – Cost of late effects

## Systematic search of costing studies for breast and lung cancer, CHD, and stroke

In order to evaluate the possible cost-effectiveness of PBT, the previously developed model was augmented to also calculate costs attributable to late effects from the UK National Health Service (NHS) perspective. A systematic literature search was performed to identify appropriate studies on which to base these costs. MEDLINE, EMBASE and Web of Science were searched from 1 January 2010 to 29 June 2021. The search strategies for each disease, are presented in below.

| **Medline and Embase** | Searches |
| --- | --- |
| 1 (breast and lung cancer) | (cancer or carcinoma or neoplasm).ti,ab. |
| 1 (coronary heart disease) | (coronary heart disease or coronary artery disease or ischaemic heart disease or ischemic heart disease).ti,ab. |
| 1 (ischemic stroke) | stroke.ti,ab. |
| 2 | cost.ti,ab. |
| 3 | (UK or NHS or United Kingdom or England or Scotland or Wales or Northern Ireland).mp. |
| 4 | 1 and 2 and 3 |
| 5 | limit 4 to yr="2010 -Current" |
| 6 | limit 5 to english language |

| **WoS** | Searches |
| --- | --- |
| 1 | TS=(cost) |
| 2 (breast and lung cancer) | (TS=(cancer)) OR TS=(carcinoma) |
| 2 (coronary heart disease) | TS=("coronary heart disease") OR TS=("coronary artery disease") OR TS=("ischaemic heart disease") OR TS=("ischemic heart disease") |
| 2 (ischemic stroke) | TS=(stroke) |
| 3 | ((((((((((TS=(UK)) OR TS=(NHS)) OR TS=(United Kingdom)) OR TS=(England)) OR TS=(English)) OR TS=(Scotland)) OR TS=(Scottish)) OR TS=(Wales)) OR TS=(Welsh)) OR TS=(Northern Ireland)) OR TS=(Northern Irish) |
| 4 | 1 and 2 and 3 |
| 5 | Index Date: 01-01-2010 – 29-07-2021 |

General inclusion/exclusion criteria were as follows:

Inclusion criteria:

- Cancer/CHD/stroke patients of any age receiving healthcare in the UK

- Studies that calculate per-patient costs

Exclusion criteria:

- Commentaries or studies that do not report patient-level costs

- Studies which only calculate prevalence-based costs from aggregated expenditure data

Ideally, the model would incorporate published estimates of the per-patient cost for a large number of years following diagnosis, or a single estimate of the lifetime cost. Recurrence and disease-related sequelae can occur many years after initial diagnosis and treatment, therefore, studies with a short time frame may not capture the full healthcare costs of a late effect. Preference was given to full articles over abstracts, and for the cancer focused literature, studies which estimated costs for both breast and lung cancer, and therefore used the same methods to measure both costs.

*Results for Cancer*

The search strategy for breast and lung cancer costs identified 6172 records. De-duplication using the reference manager’s duplicate identifier tool reduced this to 4162 record. Titles were screened for relevance to cancer, greatly reducing the number of records to 136, which were retrieved. Articles for these 136 records were sought and assessed for eligibility. In total 11 studies were found to match the inclusion/exclusion criteria above, of which two studies looked at costs for both lung and breast cancer. Hall et al (2015)(20) included costs for only the first 15 months after diagnosis, whilst Laudicella et al (2016)(21) provided costs up to 10 and 5 years after diagnosis for breast and lung cancer respectively. For this reason, these costs from Laudicella et al (2016) were used for the model. A PRISMA 2020 flow diagram of the identification process and the characteristics of the 11 studies is given in below as well as references for the studies.

| **Publication Year** | **Author** | **Site** | **Article type** | **Time frame** |
| --- | --- | --- | --- | --- |
| 2020 | Sun et al(22) | Breast | Full article | Diagnosis to 1 year |
| 2020 | Verleger et al(23) | Lung | Full article | Diagnosis through most recent visit/death |
| 2018 | Andreas et al(24) | Lung | Full article | Diagnosis to 2 year |
| 2017 | Solem et al(25) | Lung | Abstract | Diagnosis through most recent visit/death |
| 2017 | Solem et al(26) | Lung | Abstract | Diagnosis through most recent visit/death |
| 2016 | Laudicella et al(21) | Breast and Lung | Full article | Diagnosis to 10/5 years for breast/lung cancer |
| 2016 | Marti et al(27) | Breast | Full article | 3-month period 12–15 months post-diagnosis |
| 2016 | Kennedy et al(28) | Lung | Full article | Diagnosis to 1 year |
| 2015 | McGuire et al(29) | Lung | Full article | Diagnosis to 2 year |
| 2015 | Hall et al(20) | Breast and Lung | Full article | Diagnosis to 15 months |
| 2014 | Hall et al(30) | Breast | Abstract | Diagnosis to 10 years or until death if earlier |

Records identified from:

Medline (n = 1053)

EMBASE (n = 2949)

Web of Science (n = 2170)

Records removed *before screening*:

Duplicate records removed (n = 2010)

**Identification**

Records screened

(n = 4162)

Records excluded**

(n = 4026)

Reports excluded:

Editorial (n = 1)

Study of the cost treatment/diagnosis (n = 18)

Cost of trial (n = 1)

Did not include patient-specific costs (n = 18)

Non-UK setting (n = 42)

Prevalence-based or aggregate costs (n = 12)

Societal costs (n = 2)

Review (n = 6)

Not related to breast or lung cancer (n = 21)

End of life (n = 4)

Reports sought for retrieval

(n = 136)

**Screening**

Reports assessed for eligibility

(n = 136)

Studies included in review

(n = 11)

**Included**

*Results for CHD*

655 records were identified for CHD of which 316 remained after de-duplication. Screening titles and abstracts for relevance to the inclusion criteria excluded 308 records, leaving 8 sought for retrieval and full article review, which subsequently identified only two studies as meeting the requirements. Both publications were part of the same project, one focusing on methodology whilst the other focusing on presentation of the lifetime costs of stable coronary artery disease. Lifetime costs were abstracted from this later study. A PRISMA 2020 flow diagram of the identification process and the characteristics of the 8 studies is given below.

| **Publication Year** | **Author** | **Article type** | **Time frame** |
| --- | --- | --- | --- |
| 2016 | Walker et al(31) | Full article | Lifetime |
| 2016 | Asaria et al(32) | Full article | Lifetime |

Records identified from:

Medline & EMBASE (n = 398)

Web of Science (n = 257)

Records removed *before screening*:

Duplicate records removed (n = 339)

**Identification**

Records screened

(n = 316)

Records excluded**

(n = 308)

Reports sought for retrieval

(n = 8)

**Screening**

Reports excluded:

Non-CHD specific costs (n = 4)

Review (n = 2)

Reports assessed for eligibility

(n = 8)

Studies included in review

(n = 2)

**Included**

*Results for stroke*

The search strategy for stroke costs identified 1642 records, reducing to 1041 records after de-duplication. Titles were screened for relevance to costs and CHD leaving 24 records for retrieval. Articles for these 24 records were sought and assessed for eligibility, of which 8 were found to be eligible. Only 2 of the studies looked at the lifetime costs. A PRISMA 2020 flow diagram of the identification process and the characteristics of the 8 studies is given in below as well as references for the studies. The more recent study by Patel et al (2020) builds on the discrete-simulation model informed by routine registry and audit data developed in Xu et al (2018), therefore, cost estimates from the former are used in our model.

| **Publication Year** | **Author** | **Article type** | **Time frame** |
| --- | --- | --- | --- |
| 2021 | Bakhai et al(33) | Full article | 1 year post-index |
| 2020 | Patel et al(34) | Full article | Lifetime |
| 2018 | Xu et al(35) | Full article | Lifetime |
| 2015 | Ali et al(36) | Full article | Period of acute care |
| 2014 | Yiin et al(37) | Full article | 1 year |
| 2012 | Burton et al(38) | Full article | 1 year |
| 2012 | Luengo-Fernandez et al(39) | Full article | 5 year |
| 2011 | Gillespie et al(40) | Full article | Length of hospital care |

Records identified from:

Medline & EMBASE (n = 1096)

Web of Science (n = 546)

Records removed *before screening*:

Duplicate records removed (n = 601)

**Identification**

Records screened

(n = 1041)

Records excluded**

(n = 1017)

Reports sought for retrieval

(n = 24)

**Screening**

Reports excluded:

Not related to stroke (n = 11)

Population costs (n = 1)

Not related to costs (n = 2)

Non-UK setting (n = 2)

Reports assessed for eligibility

(n = 24)

Studies included in review

(n = 8)

**Included**

## Incorporation into model

*Cancer costs*

The study by Laudicella et al (2016)(21) estimated the mean NHS costs for the year 2010/11 incurred by breast and lung cancer patients from diagnosis to up to 10 and 5 years post-diagnosis respectively. Additionally these mean costs were given by age at diagnosis; younger than 65, and 65 and over. In the model, the patient age was taken into account and costs are accrued corresponding to the costs for the years since the patient transitioned from the no disease (lung/breast cancer) to diseased (lung/breast cancer) states.

*CHD costs*

Asaria et al(32) provides a single discounted lifetime mean NHS cost, based on 2011/12 prices, after diagnosis of SCAD. In the model this was applied if a patient transitioned from the no CHD to diseased state.

*Stroke costs*

Patel et al (2020) estimate age-group (<65, <75, <85) specific NHS costs (year 2014/15) for the first year following a stroke, which were accrued in our model when a patient transitioned from no ischemic stroke to diseased. The study also provides an estimate of the mean annual cost for subsequent years after the one-year post-stroke period. In our model, patients in the diseased state of the stroke submodule accrue this cost every year following the initial transition.

*Inflation of costs*

In our model costs are reported for the year 2014/15. The cancer costs and CHD costs were inflated using the Hospital and Community Health Services (HCHS) Index

# Appendix 3 – Generation of illustrative patients

The following procedure was used to create the 606 illustrative patients:

1. Starting with the data from the comparative planning study

| **Study patient no.** | **Study patient sex** | **Study patient age** | **MBD BVMAT** | **MBD PBT** | **MLD BVMAT** | **…** | **MDCCA PBT** | **CTV to heart overlap** | **…** | **CTV extension LMSCA** |
| --- | --- | --- | --- | --- | --- | --- | --- | --- | --- | --- |
| 1 | Male | 47 | NA | NA | … | … | … | 39% | … | Above |
| 2 | Female | 19 | 2.1 Gy | 1.8 Gy | … | … | … | 12% | … | Below |
| … | … | … | … | … | … | … | … | … | … | … |
| 61 | Female | 32 | 3.4 Gy | 0.4 Gy | … | … | … | …. | … | … |

1. The mean organ dose pairs for the 61 comparatively planned patients (40 female, 21 male) were used with both sex variables to give 101 notional patients (40 notional female patients due to the 21 male patients lacking breast dose and 61 notional male patients - setting mean breast dose to zero)

| No. notional patients | Notional sex | Study patient no. | ~~Study patient sex~~ | Study patient age | MBD BVMAT | … | CTV extension LMSCA |
| --- | --- | --- | --- | --- | --- | --- | --- |
| 1 | Male | 1 | ~~Male~~ | 47 | NA | … | Above |
| … | Male | … | ~~…~~ |  | … | … | … |
| 61 | Male | 61 | ~~Female~~ | 32 | 3.4 Gy | … | … |
| 62 | Female | 2 | ~~Female~~ | 19 | 2.1 Gy | … | Below |
| … | Female | … | ~~…~~ |  | … | … | … |
| 101 | Female | 61 | ~~Female~~ | 32 | 3.4 Gy | … | … |

1. Each of these 101 patients were given age at treatment inputs of 20y and 40y, giving a total of 202 notional patients with unique input variable combinations on which the model is run

| No. notional patients | Notional age | Notional sex | Study patient no. | ~~Study patient sex~~ | ~~Study patient age~~ | MBD BVMAT | … | CTV extension LMSCA |
| --- | --- | --- | --- | --- | --- | --- | --- | --- |
| 1 | 20y | Male | 1 | ~~Male~~ | ~~47~~ | NA | … | Above |
| … | … | … | … | ~~…~~ | ~~…~~ | … | … | … |
| 101 | 20y | Female | 61 | ~~Female~~ | ~~32~~ | 3.4 Gy | … | … |
| 102 | 40y | Male | 1 | ~~Male~~ | ~~47~~ | NA | … | Above |
| … | … | … | … | ~~…~~ | ~~…~~ | … | … | … |
| 202 | 40y | Female | 61 | ~~Female~~ | ~~32~~ | 3.4 Gy | … | … |

1. Finally, each of the 202 notional patients were given the three smoking statuses; never, former, and current. The final patient dataset contains 606 constructed patients

| No. notional patients | Notional smoking status | Notional age | Notional sex | Study patient no. | ~~Study patient sex~~ | ~~Study patient age~~ | MBD BVMAT | … | CTV extension LMSCA |
| --- | --- | --- | --- | --- | --- | --- | --- | --- | --- |
| 1 | Never | 20y | Male | 1 | ~~Male~~ | ~~47~~ | NA | … | Above |
| 2 | Former | … | … | … | ~~…~~ | ~~…~~ | … | … | … |
| 3 | Current | 20y | Male | 1 | ~~Male~~ | ~~47~~ | NA | … | Above |
| … | … | … | … | … | ~~..~~ | ~~…~~ | … | … | … |
| 604 | Never | 40y | Female | 61 | ~~Female~~ | 32 | 3.4 Gy | … | … |
| 605 | Former | … | … | … | ~~…~~ | ~~…~~ | … | … | … |
| 606 | Current | 40y | Female | 61 | ~~Female~~ | ~~32~~ | 3.4 Gy | … | … |

#

# Appendix 4 – Deriving the maximum additional cost of PBT over photon-based radiotherapy for PBT to be considered cost-effective

The UK’s NICE currently uses an incremental cost-effectiveness ratio (ICER) threshold in the range of £20,000 to £30,000 per quality-adjusted life year (QALY) for reimbursing in the NHS. Given an ICER threshold, and the patients expected discounted QALYs and late effects related healthcare costs, the maximum upfront cost difference between PBT and BVMAT can be calculated through rearrangement of the ICER equation as shown below. For each patient we calculate this value, under the higher £30,000 per QALY threshold.

$$ICER= \frac{{Cost}_{PBT}-{Cost}_{BVMAT}}{{QALYs}_{PBT}-{QALYs}_{BVMAT}}$$

Let,

$$k=Willingness to pay threshold$$

$$\Delta{Cost}_{RT}=The difference in upront cost of delivering PBT and BVMAT$$

$${\Delta Cost}_{Late effects}=The difference in healthcare costs from late effects$$

$$\Delta QALYs=The difference in QALYs$$

Then to be considered cost effective,

$$k>\frac{\Delta{Cost}_{RT}+{\Delta Cost}_{Late effects}}{\Delta QALYs}$$

$$\left( k*\Delta QALYs \right)+{\Delta Cost}_{Late effects}>\Delta{Cost}_{RT}$$

# Appendix 5 – Additional tables

## Table 1: Health state transitions in the ISHL sub-model

| **Transition** | **Estimate and Description** |
| --- | --- |
| **Remission to First relapse** | Yearly transition probabilities for the first 5 years after initial treatment obtained from a generalized F distribution: mu = 1.64229, sigma = -0.4577225, shape 1 = -48.84879, shape 2 = -6.897889. This corresponds to a 2.9%, 2.2%, 1.3%, 0.9%, and 0.7% yearly probability of relapse over the 5 years.(1) |
| **Remission to Cured** | Patients were considered cured after 5 years |
| **First relapse to Second relapse** | Yearly transition probabilities for the first 3 years after first relapse obtained from a log-normal distribution: log mean = 3∙715, log SD = 1∙577. This corresponds to yearly relapse probabilities of 21∙8%, 19∙1% and 15∙8% over the 3 years.(5) |
| **First relapse to Cured** | Patients were considered cured after 3 years |
| **Second relapse to Dead from HL** | Yearly transition probabilities for the first 5 years after second relapse obtained from an exponential distribution: rate = 0∙00886. This corresponds to a 10∙1% yearly probability of death from HL over the 10 years.(6) |
| **Second relapse to Cured** | Patients were considered cured after 10 years |

## Table 2: Summary of the published dose-response relationships used to estimate risks of radiation-related second cancers, CHD and stroke

| **Late toxicity** | **Excess relative risk per Gray equation** | **Input dosimetry variable** | **Relative risk** |
| --- | --- | --- | --- |
| **Breast cancer**(7,18,19) | < 19 years, ERR/Gy = 0∙257; 20-24 years, ERR/Gy = 0∙097; 25-29 years, ERR/Gy = 0∙057; 30-34 years, ERR/Gy = 0∙043; > 34 years, ERR/Gy = 0∙030 | Mean breast dose to bilateral breast tissue (MBD) | RR=1+(ERR/Gy x MBD x 1∙608†) |
| **Lung cancer**(8,9) | ERR/Gy, 0∙15 (95% CI: 0∙06-0∙39) | Mean lung dose to the whole lungs (MLD) | RR=1+(ERR/Gy x MLD x 1∙672†) |
| **Coronary heart disease**(10) | < 28 years, ERR/Gy = 0∙200 (95% CI: 0∙052-0∙070); 28-36 years, ERR/Gy = 0∙088 (95% CI: 0∙026-0∙229); >36 years, ERR/Gy = 0∙042 (95% CI: 0∙006-0∙111) | Mean heart dose to the whole heart (MHD) | RR = 1 + ERR*MHD |
| **Ischemic stroke**(16) | < 21 years, ERR/Gy = 0∙068 (95% CI: 0∙015-0∙156). 21-30 years, ERR/Gy = 0∙051 (95% CI: 0∙022-0∙088); 31-40 years, ERR/Gy = 0∙024 (95% CI: 0∙005-0∙051); > 40 years, ERR/Gy = 0∙0098 (95% CI: -0∙007-0∙032) | Mean dose to the common carotid arteries (MDCCA) | RR = 1 + ERR*MDCCA |

† Adjustment factors to allow the use of mean organ dose for predictions (rather than point dose at site of second cancer development)

## Table 3: EQ-5D values from Sullivan et al (2011)(41)

| **Type** | | **Mean utility value/decrement** | **Standard Error** |
| --- | --- | --- | --- |
| **Age band – Web table 1** | 10-19 | 0.913 | 0.004 |
|  | 20-29 | 0.905 | 0.002 |
|  | 30-39 | 0.879 | 0.002 |
|  | 40-49 | 0.837 | 0.003 |
|  | 50-59 | 0.798 | 0.004 |
|  | 60-69 | 0.774 | 0.004 |
|  | 70-79 | 0.723 | 0.005 |
|  | 80+ | 0.657 | 0.008 |
| **Disease decrement from ICD9 diagnosis** | Transplant (Autologous stem-cell transplant) | -0.142 | 0.077 |
|  | Breast cancer | -0.019 | 0.014 |
|  | Lung cancer | -0.119 | 0.043 |
|  | Coronary heart disease | -0.037 | 0.026 |
|  | Ischemic stroke | -0.073 | 0.009 |

## Table 4: Proportions of people who have never smoked, ex-smokers and smokers from the 2012 General Lifestyle Survey

| **Age band** | **Sex** | **Proportion never smokers** | **Proportion of ex-smokers** | **Proportion of smokers** |
| --- | --- | --- | --- | --- |
| **15-19** | male | 0.80669 | 0.0223 | 0.171 |
| **20-24** |  | 0.59302 | 0.07752 | 0.32946 |
| **25-29** |  | 0.53276 | 0.1339 | 0.33333 |
| **30-34** |  | 0.53511 | 0.13801 | 0.32688 |
| **35-39** |  | 0.5426 | 0.19955 | 0.25785 |
| **40-44** |  | 0.53157 | 0.1833 | 0.28513 |
| **45-49** |  | 0.54409 | 0.22326 | 0.23265 |
| **50-54** |  | 0.51454 | 0.22595 | 0.25951 |
| **55-59** |  | 0.46042 | 0.30833 | 0.23125 |
| **60-64** |  | 0.41586 | 0.38685 | 0.19729 |
| **65-69** |  | 0.40319 | 0.4511 | 0.14571 |
| **70-74** |  | 0.40642 | 0.47326 | 0.12032 |
| **80+** |  | 0.42834 | 0.50082 | 0.07084 |
| **15-19** | female | 0.77255 | 0.01961 | 0.20784 |
| **20-24** |  | 0.57713 | 0.09043 | 0.33245 |
| **25-29** |  | 0.59319 | 0.12425 | 0.28257 |
| **30-34** |  | 0.58681 | 0.17188 | 0.24132 |
| **35-39** |  | 0.60304 | 0.16047 | 0.23649 |
| **40-44** |  | 0.59304 | 0.17913 | 0.22783 |
| **45-49** |  | 0.61191 | 0.14982 | 0.23827 |
| **50-54** |  | 0.587 | 0.19503 | 0.21797 |
| **55-59** |  | 0.55028 | 0.26376 | 0.18596 |
| **60-64** |  | 0.60204 | 0.2466 | 0.15136 |
| **65-69** |  | 0.51705 | 0.30303 | 0.17992 |
| **70-74** |  | 0.62697 | 0.24944 | 0.1236 |
| **80+** |  | 0.6629 | 0.25792 | 0.07919 |

## Table 5: Relative risk and 95% confidence interval for lung cancer, CHD, and stroke used to adjust the population incidence rates by smoking status

| **Disease** | **Gender** | **Age** | **Relative risk and 95% confidence interval** | |
| --- | --- | --- | --- | --- |
|  | | | **Current Smokers** | **Former Smoker** |
| **Lung cancer**(42) | Male and female | All | 10.92 (8.28–14.40) | 3.85 (2.77–5.34) |
| **CHD**(43) | Male | 35–64 | 3.18 (2.34–4.33) | 1.59 (1.11–2.27) |
|  | Male | 65 | 1.96 (1.62–2.37) | 1.16 (1.01–1.34) |
|  | Female | 35–64 | 3.93 (2.56–6.05 | 1.48 (0.82–2.64) |
|  | Female | 65 | 1.95 (1.60–2.37) | 1.37 (1.18–1.58) |
| **Stroke**(44) | Male | All | 1.57 (1.49–1.88) | 1.08 (1.03–1.13) |
|  | Female | All | 1.83 (1.58–2.12) | 1.17 (1.12–1.22) |

## Table 6: The costs of breast and lung cancer

|  | **Breast cancer (£)** | | **Lung cancer (£)** | |
| --- | --- | --- | --- | --- |
| **Time period** | **<65y** | **≥65y** | **<65y** | **≥65y** |
| **Year 1** | 12343 | 8653 | 13426 | 10068 |
| **Year 2** | 4084 | 2972 | 5044 | 4800 |
| **Year 3** | 2417 | 2522 | 4446 | 4383 |
| **Year 4** | 1980 | 2536 | 2967 | 3739 |
| **Year 5** | 1897 | 2429 | 2834 | 3381 |
| **Year 6** | 1828 | 2469 | - | - |
| **Year 7** | 1621 | 2356 | - | - |
| **Year 8** | 1591 | 2382 | - | - |
| **Year 9** | 1462 | 2530 | - | - |

## Table 7: The costs of CHD and stroke

|  | **Stroke (£)** | | **Coronary heart disease (£)** |
| --- | --- | --- | --- |
| **Upper age** | **Incidence cost** | **Prevalence cost** | **Life time cost** |
| 64 | 12770 | 5169 | 24038 |
| 74 | 15577 | 5761 |  |
| 84 | 20011 | 10753 |  |
| 85 | 22961 | 9873 |  |

## Table 8: Difference in QALYs, health care costs and maximum additional upfront cost of PBT to be considered cost-effective at £30,000 threshold

| **Discount** | **Sex** | **Age** | **QALYs** | **Costs** | **Maximum cost** |
| --- | --- | --- | --- | --- | --- |
| Undiscounted | Female | Never smokers, Mean (range) | | | |
|  |  | 20 | 0.099 -0.05 to 0.491 | 357.234 -1630 to 1692 | - |
|  |  | 40 | 0.067 -0.019 to 0.254 | 93.913 -668 to 635 | - |
|  |  | Former smoker, Mean (range) | | | |
|  |  | 20 | 0.228 -0.079 to 0.819 | 329.063 -1733 to 1956 | - |
|  |  | 40 | 0.227 -0.038 to 0.669 | -152.278 -1401 to 536 | - |
|  |  | Current smoker, Mean (range) | | | |
|  |  | 20 | 0.54 -0.192 to 1.553 | 839.099 -1098 to 2602 | - |
|  |  | 40 | 0.584 -0.147 to 1.501 | -329.27 -1784 to 746 | - |
|  | Male | Never smokers, Mean (range) | | | |
|  |  | 20 | 0.062 -0.196 to 0.43 | 629.151 -1693 to 4037 | - |
|  |  | 40 | 0.072 -0.054 to 0.273 | 209.879 -609 to 1525 | - |
|  |  | Former smoker, Mean (range) | | | |
|  |  | 20 | 0.243 -0.235 to 1.038 | 632.69 -1757 to 3878 | - |
|  |  | 40 | 0.271 -0.082 to 0.888 | 30.651 -1032 to 1356 | - |
|  |  | Current smoker, Mean (range) | | | |
|  |  | 20 | 0.596 -0.342 to 2.171 | 1250.805 -1583 to 4598 | - |
|  |  | 40 | 0.669 -0.205 to 2.146 | -39.878 -1586 to 1839 | - |
| 3.5% discount | Female | Never smokers, Mean (range) | | | |
|  |  | 20 | 0.018 -0.008 to 0.086 | -5.297 -554 to 221 | 544 -392 to 3128 |
|  |  | 40 | 0.02 -0.005 to 0.074 | -8.145 -333 to 184 | 620 -158 to 2509 |
|  |  | Former smoker, Mean (range) | | | |
|  |  | 20 | 0.037 -0.01 to 0.135 | -28.032 -605 to 294 | 1128 -403 to 4655 |
|  |  | 40 | 0.068 -0.01 to 0.202 | -106.242 -621 to 149 | 2135 -357 to 6622 |
|  |  | Current smoker, Mean (range) | | | |
|  |  | 20 | 0.087 -0.026 to 0.252 | -6.984 -613 to 385 | 2608 -960 to 8125 |
|  |  | 40 | 0.181 -0.045 to 0.471 | -260.119 -965 to 183 | 5677 -1519 to 15101 |
|  | Male | Never smokers, Mean (range) | | | |
|  |  | 20 | 0.009 -0.03 to 0.065 | 91.056 -365 to 619 | 189 -1314 to 2338 |
|  |  | 40 | 0.021 -0.017 to 0.08 | 54.39 -237 to 459 | 575 -727 to 2546 |
|  |  | Former smoker, Mean (range) | | | |
|  |  | 20 | 0.036 -0.036 to 0.157 | 69 -571 to 636 | 1024 -1533 to 5102 |
|  |  | 40 | 0.081 -0.023 to 0.265 | -29.652 -465 to 412 | 2452 -927 to 8387 |
|  |  | Current smoker, Mean (range) | | | |
|  |  | 20 | 0.096 -0.06 to 0.351 | 113.575 -774 to 825 | 2766 -2447 to 11039 |
|  |  | 40 | 0.213 -0.067 to 0.686 | -133.615 -949 to 584 | 6538 -2313 to 21539 |

# Appendix 6 – Additional Figure

## Figure 1: Box plots of the estimated difference in undiscounted quality-adjusted life years between PBT and VMAT for former smokers across the four prognostic markers


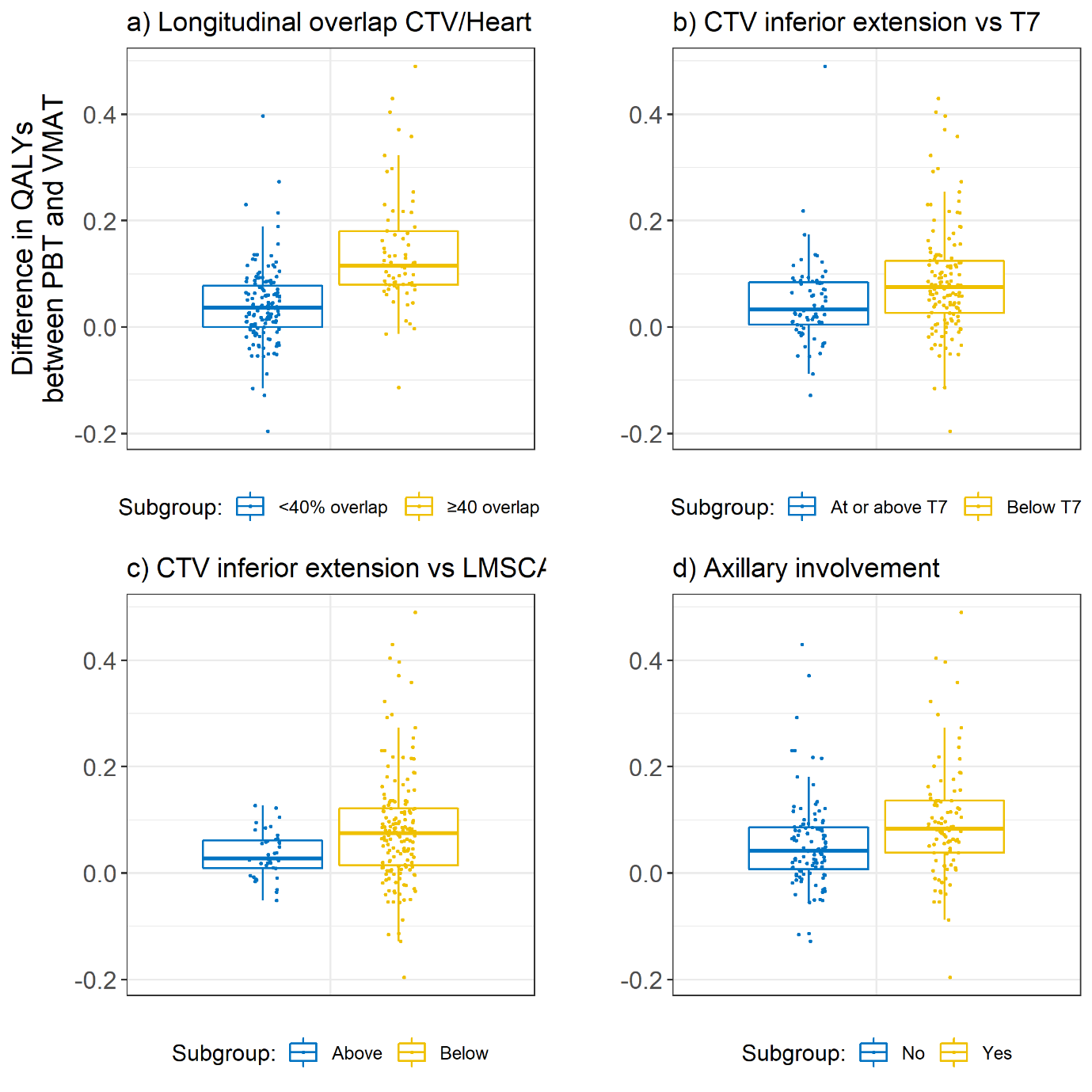


# References

1. André MPE, Girinsky T, Federico M, Reman O, Fortpied C, Gotti M, et al. Early Positron Emission Tomography Response–Adapted Treatment in Stage I and II Hodgkin Lymphoma: Final Results of the Randomized EORTC/LYSA/FIL H10 Trial. J Clin Oncol. 2017 Mar 14;35(16):1786–94.

2. Guyot P, Ades A, Ouwens MJ, Welton NJ. Enhanced secondary analysis of survival data: reconstructing the data from published Kaplan-Meier survival curves. BMC Med Res Methodol. 2012 Feb 1;12(1):9.

3. Baio G. survHE: Survival Analysis for Health Economic Evaluation and Cost-Effectiveness Modeling. J Stat Softw. 2020 Oct 7;95(1):1–47.

4. Lagerlöf I, Holte H, Glimelius I, Björkholm M, Enblad G, Erlanson M, et al. No excess long-term mortality in stage I-IIA Hodgkin lymphoma patients treated with ABVD and limited field radiotherapy. Br J Haematol. 2020 Mar 1;188(5):685–91.

5. Moskowitz CH, Nademanee A, Masszi T, Agura E, Holowiecki J, Abidi MH, et al. Brentuximab vedotin as consolidation therapy after autologous stem-cell transplantation in patients with Hodgkin’s lymphoma at risk of relapse or progression (AETHERA): a randomised, double-blind, placebo-controlled, phase 3 trial. The Lancet. 2015 May 9;385(9980):1853–62.

6. Bair SM, Strelec L, Nagle SJ, Nasta SD, Landsburg DJ, Mato AR, et al. Outcomes of patients with relapsed/refractory Hodgkin lymphoma progressing after autologous stem cell transplant in the current era of novel therapeutics: A retrospective analysis. Am J Hematol. 2017;92(9):879–84.

7. Travis LB, Hill DA, Dores GM, Gospodarowicz M, van Leeuwen FE, Holowaty E, et al. Breast Cancer Following Radiotherapy and Chemotherapy Among Young Women With Hodgkin Disease. JAMA. 2003 Jul 23;290(4):465–75.

8. Gilbert ES, Stovall M, Gospodarowicz M, van Leeuwen FE, Andersson M, Glimelius B, et al. Lung Cancer after Treatment for Hodgkin’s Disease: Focus on Radiation Effects. Radiat Res. 2003 Feb 1;159(2):161–73.

9. Travis LB, Gospodarowicz M, Curtis RE, Aileen Clarke E, Andersson M, Glimelius B, et al. Lung Cancer Following Chemotherapy and Radiotherapy for Hodgkin’s Disease. JNCI J Natl Cancer Inst. 2002 Feb 6;94(3):182–92.

10. van Nimwegen FA, Schaapveld M, Cutter DJ, Janus CPM, Krol ADG, Hauptmann M, et al. Radiation Dose-Response Relationship for Risk of Coronary Heart Disease in Survivors of Hodgkin Lymphoma. J Clin Oncol. 2015 Nov 16;34(3):235–43.

11. Scarborough P, Harrington RA, Mizdrak A, Zhou LM, Doherty A. The Preventable Risk Integrated ModEl and Its Use to Estimate the Health Impact of Public Health Policy Scenarios. Mastrangelo G, editor. Scientifica. 2014 Sep 25;2014:748750.

12. Briggs ADM, Cobiac LJ, Wolstenholme J, Scarborough P. PRIMEtime CE: a multistate life table model for estimating the cost-effectiveness of interventions affecting diet and physical activity. BMC Health Serv Res. 2019 Jul 16;19(1):485.

13. Kent S, Aveyard P, Astbury N, Mihaylova B, Jebb SA. Is Doctor Referral to a Low-Energy Total Diet Replacement Program Cost-Effective for the Routine Treatment of Obesity? Obesity. 2019 Mar 1;27(3):391–8.

14. Hunt D, Knuchel-Takano A, Jaccard A, Bhimjiyani A, Retat L, Selvarajah C, et al. Modelling the implications of reducing smoking prevalence: the public health and economic benefits of achieving a ‘tobacco-free’ UK. Tob Control. 2018 Mar 1;27(2):129–35.

15. Hiding in plain sight: Treating tobacco dependency in the NHS [Internet]. RCP London. 2018 [cited 2021 Aug 31]. Available from: https://www.rcplondon.ac.uk/projects/outputs/hiding-plain-sight-treating-tobacco-dependency-nhs

16. De Bruin ML, Dorresteijn LDA, van’t Veer MB, Krol ADG, van der Pal HJ, Kappelle AC, et al. Increased Risk of Stroke and Transient Ischemic Attack in 5-Year Survivors of Hodgkin Lymphoma. JNCI J Natl Cancer Inst. 2009 Jul 1;101(13):928–37.

17. Maraldo MV, Lundemann M, Vogelius IR, Specht L. A new method to estimate doses to the normal tissues after past extended and involved field radiotherapy for Hodgkin lymphoma. Radiother Oncol. 2015 Feb 1;114(2):206–11.

18. Swerdlow AJ, Cooke R, Bates A, Cunningham D, Falk SJ, Gilson D, et al. Breast Cancer Risk After Supradiaphragmatic Radiotherapy for Hodgkin’s Lymphoma in England and Wales: A National Cohort Study. J Clin Oncol. 2012 Aug 1;30(22):2745–52.

19. Schaapveld M, Aleman BMP, van Eggermond AM, Janus CPM, Krol ADG, van der Maazen RWM, et al. Second Cancer Risk Up to 40 Years after Treatment for Hodgkin’s Lymphoma. N Engl J Med. 2015 Dec 23;373(26):2499–511.

20. Hall PS, Hamilton P, Hulme CT, Meads DM, Jones H, Newsham A, et al. Costs of cancer care for use in economic evaluation: a UK analysis of patient-level routine health system data. Br J Cancer. 2015 Mar;112(5):948–56.

21. Laudicella M, Walsh B, Burns E, Smith PC. Cost of care for cancer patients in England: evidence from population-based patient-level data. Br J Cancer. 2016 May;114(11):1286–92.

22. Sun L, Cromwell D, Dodwell D, Horgan K, Gannon MR, Medina J, et al. Costs of Early Invasive Breast Cancer in England Using National Patient-Level Data. Value Health. 2020 Oct 1;23(10):1316–23.

23. Verleger K, Penrod JR, Daumont MM, Solem C, Luo L, Macahilig C, et al. Costs and cost drivers associated with non-small-cell lung cancer patients who received two or more lines of therapy in Europe. Clin Outcomes Res. 2020;12((Verleger) Pharmerit International, Berlin, Germany):23–33.

24. Andreas S, Chouaid C, Danson S, Siakpere O, Hoffmann H, Potter V, et al. Economic burden of resected (stage IB-IIIA) non-small cell lung cancer in France, Germany and the United Kingdom: A retrospective observational study (LuCaBIS). Lung Cancer. 2018;124((Andreas) Lungenfachklinik Immenhausen, Krs. Kassel and Universitatsmedizin Gottingen, Gottingen 37075, Germany):298–309.

25. Solem CT, Luo L, Penrod JR, Lees M, Macahilig C, Verleger K, et al. Cost drivers of lung cancer care: Results from a retrospective chart review of pretreated advanced nsclc patients in europe. Value Health. 2017;20(9):A431–2.

26. Solem CT, Luo L, Penrod JR, Lees M, Macahilig C, Verleger K, et al. Real-world health care resource utilization and related costs among patients who received at least two lines of treatment for advanced NSCLC in England, the Netherlands, and Sweden. Value Health. 2017;20(9):A431.

27. Marti J, Hamilton P, Hulme CT, Hall PS, Jones H, Velikova G, et al. The economic burden of cancer in the UK: A study of survivors treated with curative intent. Psychooncology. 2016;25(1):77–83.

28. Kennedy MPT, Callister MEJ, Hall PS. Healthcare costs associated with lung cancer diagnosed at emergency hospitalisation. Lung Cancer. 2016;91(Supplement 1):S38–9.

29. McGuire A, Martin M, Lenz C, Sollano JA. Treatment cost of non-small cell lung cancer in three European countries: Comparisons across France, Germany, and England using administrative databases. J Med Econ. 2015;18(7):525–32.

30. Hall P, Newsham A, Hall G, Glaser A, Walkington L. Costs of hospital care over ten years from diagnosis of early breast cancer in England. Eur J Cancer. 2014;50(SUPPL. 2):S79–80.

31. Walker S, Asaria M, Manca A, Palmer S, Gale CP, Shah AD, et al. Long-term healthcare use and costs in patients with stable coronary artery disease: a population-based cohort using linked health records (CALIBER). Eur Heart J - Qual Care Clin Outcomes. 2016 Apr 1;2(2):125–40.

32. Asaria M, Walker S, Palmer S, Gale CP, Shah AD, Abrams KR, et al. Using electronic health records to predict costs and outcomes in stable coronary artery disease. Heart. 2016 May 15;102(10):755–62.

33. Bakhai A, Petri H, Vahidnia F, Wolf C, Ding Y, Sculpher M, et al. Real-world data on the incidence, mortality, and cost of ischaemic stroke and major bleeding events among non-valvular atrial fibrillation patients in England. J Eval Clin Pract. 2021;27(1):119–33.

34. Patel A, Berdunov V, Quayyum Z, King D, Knapp M, Wittenberg R. Estimated societal costs of stroke in the UK based on a discrete event simulation. Age Ageing. 2020 Feb 27;49(2):270–6.

35. Xu XM, Vestesson E, Paley L, Hoffman A, Desikan A, Wolfe CDA, et al. The economic burden of stroke care in England, Wales and Northern Ireland: Using a national stroke register to estimate and report patient-level health economic outcomes in stroke. Eur Stroke J. 2018;3(1):82–91.

36. Ali AN, Howe J, Abdel-Hafiz A. Cost of acute stroke care for patients with atrial fibrillation compared with those in sinus rhythm. PharmacoEconomics. 2015;33(5):511–20.

37. Yiin GSC, Howard DPJ, Paul NLM, Li L, Luengo-Fernandez R, Bull LM, et al. Age-specific incidence, outcome, cost, and projected future burden of atrial fibrillation-related embolic vascular events: A population-based study. Circulation. 2014;130(15):1236–44.

38. Burton TM, Lacey M, Liu F, Yu Y, Sander S, Monsalvo ML, et al. One-year follow-up healthcare costs of patients hospitalized for transient ischemic attack or ischemic stroke and discharged with aspirin plus extended-release dipyridamole or clopidogrel. J Med Econ. 2012;15(6):1217–25.

39. Luengo-Fernandez R, Gray AM, Rothwell PM. A population-based study of hospital care costs during 5 years after transient ischemic attack and stroke. Stroke. 2012;43(12):3343–51.

40. Gillespie J, McClean S, Scotney B, Garg L, Barton M, Fullerton K. Costing hospital resources for stroke patients using phase-type models. Health Care Manag Sci. 2011;14(3):279–91.

41. Sullivan PW, Slejko JF, Sculpher MJ, Ghushchyan V. Catalogue of EQ-5D Scores for the United Kingdom. Med Decis Making. 2011 Mar 21;31(6):800–4.

42. Jayes L, Haslam PL, Gratziou CG, Powell P, Britton J, Vardavas C, et al. SmokeHaz: Systematic Reviews and Meta-analyses of the Effects of Smoking on Respiratory Health. Chest. 2016 Jul;150(1):164–79.

43. Rostron B. Smoking-Attributable Mortality by Cause in the United States: Revising the CDC’s Data and Estimates. Nicotine Tob Res. 2013 Jan 1;15(1):238–46.

44. Peters SAE, Huxley RR, Woodward M. Smoking as a Risk Factor for Stroke in Women Compared With Men. Stroke. 2013 Oct 1;44(10):2821–8.

45. Jones DA, Candio P, Shakir R, Ntentas G, Ramroth J, Gray AM, et al. Informing radiotherapy decisions in stage I/IIa Hodgkin lymphoma: modeling life expectancy using radiation dosimetry. Blood Adv. 2022 Feb 8;6(3):909–19.
